# Carbon elimination from silicon kerf: Thermogravimetric analysis and mechanistic considerations

**DOI:** 10.1038/srep40535

**Published:** 2017-01-18

**Authors:** Miguel Vazquez-Pufleau, Tandeep S. Chadha, Gregory Yablonsky, Pratim Biswas

**Affiliations:** 1 Aerosol and Air Quality Research Laboratory Department of Energy, Environmental & Chemical Engineering Washington University in St. Louis St. Louis, MO 63130, USA; 2 Parks College Department of Chemistry Saint Louis University St. Louis, MO 63103, USA

## Abstract

40% of ultrapure silicon is lost as kerf during slicing to produce wafers. Kerf is currently not being recycled due to engineering challenges and costs associated with removing its abundant impurities. Carbon left behind from the lubricant remains as one of the most difficult contaminants to remove in kerf without significant silicon oxidation. The present work enables to better understand the mechanism of carbon elimination in kerf which can aid the design of better processes for kef recycling and low cost photovoltaics. In this paper, we studied the kinetics of carbon elimination from silicon kerf in two atmospheres: air and N_2,_ under a regime of no-diffusion-limitation. We report the apparent activation energy in both atmospheres using three methods: Kissinger, and two isoconversional approaches. In both atmospheres, a bimodal apparent activation energy is observed, suggesting a two stage process. A reaction mechanism is proposed in which (a) C-C and C-O bond cleavage reactions occur in parallel with polymer formation; (b) at higher temperatures, this polymer fully degrades in air but leaves a tarry residue in N_2_ that accounts for about 12% of the initial total carbon.

Silicon (Si) is the main semiconductor used in electronic and solar industries. Despite Si abundance in the earth’s crust, ultrapure silicon production is expensive as it goes through a myriad of industrial refining processes. These well-established processes^1^, developed and optimized for microchip needs, provide superior quality and purity at the cost of being highly energy intensive and wasteful, in that 68% of the initial silicon is converted to waste during the refining steps^2^. Microchips need only a few grams of silicon per chip, which comprise less than 1% of the final microchip cost. However, producing a solar module requires more than 400 g of silicon per m^2^, and silicon wafers add up to more than 45% of the total cost for installed modules^1^. This shows how silicon refining inefficiencies directly translate into high cost of solar modules, hindering its large scale implementation. Therefore, efficiency improvements in silicon manufacture are highly relevant.

A key point for improvement in ultrapure silicon processing is the production of wafers, consisting of slicing silicon ingots, which wastes 45% of the ultrapure material. Efforts such as ribbon technologies to avoid the need of axial slicing have been suggested^3^. Nevertheless, traditional wafer slicing techniques have shown higher efficiencies^3^,^4^, more reliability^5^ and better return on investment of the final solar panel^6^ compared to ribbon wafers. Thus, abrasive slicing from mono and polycrystalline ingots is still the dominant technology^2^. Wafers so produced are sliced from ingots using fixed abrasive or slurry methods^7^ that unavoidably produce silicon sawing dust (kerf) impregnated in lubricant.

Silicon carbide (SiC) was traditionally used in slurry slicing techniques, and its removal was the main concern in previous studies of kerf recycling^8^,^9^,^10^,^11^. However, recent advances in understanding of silicon ductile behavior in a narrow range of cutting speeds^12^ have enabled diamond wire sawing to produce better results than slurry slicing^13^. Further optimization of diamond wire slicing is still an ongoing effort^14^. Diamond wire kerf is a complex industrial residue containing numerous metallic contaminants^15^ and organics from the polymeric lubricant used during slicing. The exact composition of this lubricant is protected by proprietary rights. However, it is known to be mainly composed of polyethylene glycol (PEG)^16^ mass average molar mass (M_w_) around 400–500 or combinations of PEG with diethylene glycol (DEG)^17^. To date, a few authors have reported methodologies for recycling kerf produced during diamond wire slicing. Their results point to the need for efficient removal of carbon. Tomono *et al*.^16^ reacted kerf with bromine forming bromosilanes for further distillation. Unfortunately, the carbon content in kerf reacted to form brominated organic compounds that behaved azeotropically and could not be separated via distillation. Dhamrin *et al*.^17^, on the other hand, melted kerf to make solar panels and showed the devastating effect that carbon contamination has on the efficiency of the so formed devices. Finally, Maeda *et al*.^18^ reported improvements in solar panel efficiency from recycled kerf by milling kerf and heating it in inert gas to remove carbon.

Previous efforts by Vazquez-Pufleau *et al*.^19^ demonstrated that carbon elimination from kerf was feasible using a furnace aerosol reactor (FuAR). In air, at high temperatures and low residence times, total carbon (TC) measurements below detection limits were achieved. To design an industrial reactor with more certainty, it is important to better understand the process and the kinetics of carbon elimination in a FuAR, including accurate activation energy (E_A_) values for the full range of conversion. To the best of our knowledge, the kinetics for the removal of organics for a metal recycling and particularly for silicon kerf, as well as the mechanistic understanding of the reaction paths, have not been systematically studied. Both will be required for industrial scale up of kerf recycling processes.

The most important parameter for determining the kinetic behavior of a reacting system with Arrhenius behavior is the E_A_^20^. The k_0_ parameter, or pre-exponential factor, can then be determined via compensation effect^21^, which depends on the reaction model employed. However, the E_A_ remains unaffected even if the kinetic model employed is incorrectly chosen. Therefore, the focus of kinetics has historically been E_A_ determinations^20^. The Kissinger method is a simple and well known technique to obtain E_A_, but this value is determined based on limited information, i.e. the peak values of the reaction rate. The values obtained are more meaningful after comparing and contrasting with isoconversional analysis, which provides reliable E_A_ values for the entire range of conversion^22^. Reliable E_A_ for the entire range of conversion is needed because a single value of E_A_ is not enough to describe the full process for polymeric decomposition, as the apparent E_A_ might be different from the intrinsic E_A_ due to competition among radical polymerization, homolysis and volatilization^23^.

In general, the elimination of residual polymer contamination from powders has not been widely studied, even less so in the case of kerf. Nevertheless, kinetic studies for pure PEG have been executed experimentally^23^,^24^,^25^,^26^,^27^,^28^,^29^ and theoretically^30^. A detail discussion on the PEG literature is provided in the supplementary section. The reported PEG literature needs to be analyzed carefully to extrapolate the findings under ideal conditions to conditions of real kerf recycling systems full of other contaminants. As Lin *et al*.^31^ have observed, the presence of nanoparticles significantly alters the degradation kinetics for PEG which follows a different degradation process when combined with Ni and Al nanoparticles. Ni accelerated the decomposition whereas Al was a retardant at the beginning but accelerated the decomposition at a later stage. This indicates that the kinetics and mechanism of removal of slicing lubricant (composed of PEG and additives) from kerf (made of silicon nanoparticles and metallic contaminants) might differ compared to pure PEG degradation.

In this work, based on thermogravimetric analysis (TGA) results, we determine the activation energy (E_A_) of carbon removal in kerf using the Kissinger method and Ozawa-Flynn-Wall (OFW) and Kissinger-Akahira-Sunose (KAS) isoconversional analysis. Additionally, we present characterization results from samples thermally treated by TGA in either air or N_2_ using Fourier transformed infrared (FTIR) spectroscopy, gas chromatography mass spectroscopy (GCMS), total carbon (TC) determination, Brunauer–Emmett–Teller (BET) surface area measurement and scanning electron microscopy (SEM). At the end, we provide an overall reaction mechanism for the elimination of carbon from kerf based on the instrumental evidence.

## Results and Discussion

Kerf was initially characterized by BET, and had a surface area of 9.6 m^2^/g, a pore volume of 0.052 cm^3^/g and a pore radius of 15.6 Å, proving its large surface area and flake-like morphology. In this section the results of each technique are presented separately followed by a brief discussion of direct implications for the individual instrumental observations summarized in Table 1. Finally, in the mechanism section, the implications for all results and individual instrument discussions are combined. Under the complexity of kerf composition and carbon elimination kinetics, a qualitative reaction mechanism is provided.

### Total carbon content

**Figure 1:**
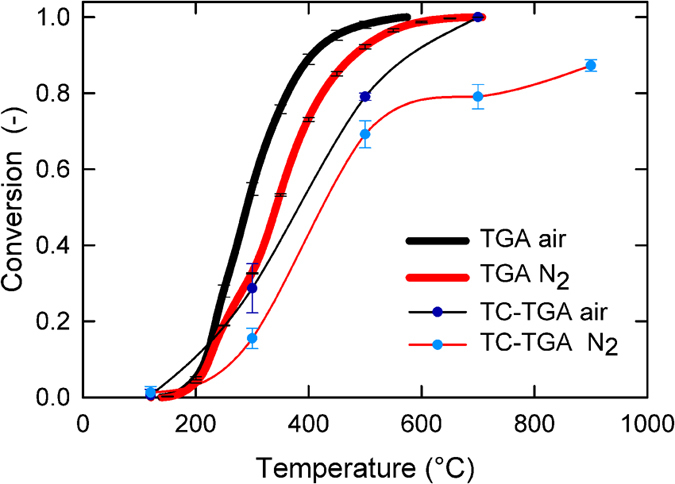
Volatiles evolution from kerf using a heating rate of 20 °C/min. Conversion is calculated by weight loss from TGA and total carbon (TC) of the remaining sample in air and in N_2_ atmospheres. Error bars indicate the standard deviation.

The conversion of carbon calculated using traditional TGA weight loss method was compared with a TGA-TC method (using TGA and stopping the run to determine its remaining total carbon (TC) content at various temperatures). The heating rate chosen of 20 °C/min is sufficiently high to emulate real aerosol reactor conditions and at the same time, low enough to guarantee mass and heat transfer. Thus, providing an accurate thermogravimetric reading. Figure 1 shows that the conversion using the two methods differs. A discussion of differences in conversion using TGA and TC is provided in the supplementary section.

### Gas chromatography mass spectroscopy

**Figure 2:**
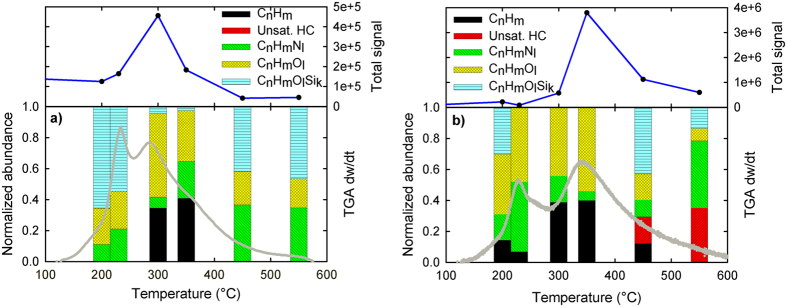
Elemental characterization of the volatiles using GCMS. Unsat. HC stands for CnHm compounds with m ~ n. Samples were extracted using a manual syringe at the indicated TGA temperatures and injected into the GCMS column. Plots (**a**) air and (**b**) N_2_ show the total signal measured from MS (upper) and the relative abundance for various groups of compounds (lower). On gray the TGA rate of weight loss as a function of temperature (lower).

Based on their chemical composition, detected peaks were classified into different categories as: C_n_H_m_ (aliphatic compounds containing only carbon and hydrogen with m ~ 2n), Unsat. HC (unsaturated hydrocarbons C_n_H_m_ with m ~ n), C_n_H_m_N_l_, C_n_H_m_O_l_ and C_n_H_m_O_l_Si_k_. The chromatogram area corresponding to all components assigned to each category were summed and normalized with respect to the total area, a relative abundance value was assigned for each category. See Fig. 2(a) for samples treated in air and Fig. 2(b) for samples treated in N_2_. A discussion follows for each atmosphere based on observations for each category except for C_n_H_m_O_l_Si_k_, whose consistent presence in most samples is explained in a separate paragraph.

In Fig. 2(a), for samples treated in air, GCMS shows that the highest rate of all volatiles formation occurs around 300 °C and no unsaturated C_n_H_m_ compounds are observed at any temperature. C_n_H_m_O_l_ compounds seem to be present in the same ratio (around 20%) at all temperatures except at the peak of volatile formation where its presence becomes more abundant. Elimination of nitrogenated compounds peak at 450 °C, showing that they are more difficult to remove than oxygenated compounds or aliphatic compounds that have an absolute peak at 300 °C.

Figure 2(b) summarizes the GCMS data for samples treated in N_2_ atmosphere. There, the peak of volatiles formation occurs at a higher temperature than in air; at about 350 °C and, beyond this temperature, a significant amount of unsaturated compounds gets released. C_n_H_m_O_l_ compounds are abundant at the beginning, but their abundance drops after 350 °C. A significant fraction of saturated compounds is visible at 450 °C, and at 550 °C all of the C_n_H_m_ compounds are already saturated. This can be interpreted as the lack of available O_2_ causes any remaining polymer in kerf to undergo saturation reactions, leaving a carbonaceous residue that is hard to pyrolyze (tar). This agrees with the TC results from Fig. 1, where not all the carbon can be eliminated. In contrast, in the air atmosphere, the availability of O_2_ permits virtually full polymer decomposition into volatiles and thus no saturated C_n_H_m_ compounds are formed. Nitrogenated compounds in N_2_ atmosphere become more abundant in terms of total signal as temperatures rise. Based on XPS evidence (See supplementary information), the nitrogen source of the nitrogenated compounds is the lubricant itself. Finally, the total GCMS signal for the pyrolysis is 6 times more intense than the one for air atmosphere (combustion) indicating that, during the combustion process, a significant fraction of volatiles fully reacts into CO_2_.

During TGA thermal operations, siloxanes seem always to be present in trace amounts. This is due to the presence of silicon nanoparticles in contact with the lubricant. Its total abundance seems to be constant, but due to large differences in the abundance of other compounds, its fraction is highly diluted on the peaks of signal and conversely overshadows other components especially at the beginning of the TGA thermal run and at the end when most of the organics have already been released.

### Fourier transformed infrared spectroscopy

**Figure 3:**
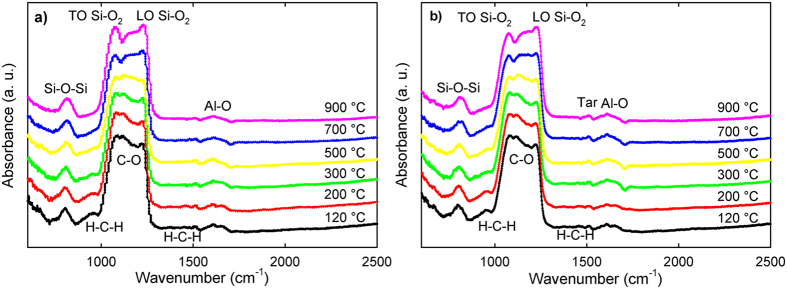
FTIR for kerf thermally treated at various temperatures (**a**) in air and (**b**) in N_2_. Peaks are labeled based on the literature.

The results of FTIR for different temperatures are displayed in Fig. 3(a) for kerf treated in air atmosphere and Fig. 3(b) for kerf treated in N_2_ atmosphere. The peak assignment with references is presented in Supplementary Table 1. The peaks can be classified into molecular motion of three groups: silicon and SiO_x_, organics, and Al. Insights into sample composition are gained through analyzing the molecular motion characteristics of the most important peaks.

Regarding silicon, the peak at ~1230 cm^−1^ is attributed to the silicon longitudinal optical (LO) mode. The peak at ~1050 cm^−1^ is attributed to transverse optical (TO) mode of the silicon asymmetric stretching vibrations^32^. It is well known that the exact peak of TO in cm^−1^ is proportional to the thickness of the alpha-SiO_2_ layer with an interface region of about 1.6 nm and corresponds to a simplified chemical formula of SiO^33^. Both the outer layer of SiO_2_ and the interface with Si include effects such as compressive stress and roughness that change the LO and TO mode. Furthermore, the peak intensity of TO and LO is directly correlated with the degree of silicon oxidation^34^. In our case TO for air is evidently larger than the one for N_2_, evidencing a higher degree of oxidation in the first scenario.

Organic peaks are small due to their low mass fraction in the sample. Before thermal treatment, total organics account for 4% of the sample, TC is 1.3%. After thermal treatment, TC approaches 0% for air and 0.15% in N_2_, imposing instrumental detection challenges. The peak at 960 cm^−1^ corresponds to hydrocarbon CH_2_ motion in the C axis^25^. A C-O stretching characteristic peak^25^ appears at 1149 cm^−1^ in the region between TO and LO altering its shape. CH_2_ scissoring is attributed to the peak at 1460 cm^−1 ^25. Depending on the molecular weight (MW) of PEG, a difference of up to 10 cm^−1^ in the peaks of characteristic bonds can be expected^35^. Finally, in the region between 1300 and 1600 cm^−1^ several peaks are observed at low temperatures. These peaks are attributed to stretching and bending of modes of aromatic hydrocarbons^36^. Most of these peaks disappear at high temperatures, indicating decomposition. However, above 700 °C in N_2_ atmosphere, a small differentiated peak can be observed that does not appear in air atmosphere. This peak is attributed to aromatic hydrocarbons^37^ that are believed to be the tarry residue following pyrolysis of the polymer mixture. This is in agreement with TC measurements.

The third most abundant contaminant in kerf after oxygen and carbon, see Table 2, is aluminum (0.3%). The peak at 1612 cm^−1^ of the kerf sample is similar for both atmospheres and is assigned to be due to Al_2_-O_3_ vibration^38^. It originates from the beam supporting the ingot during slicing.

### TGA kinetics

**Figure 4:**
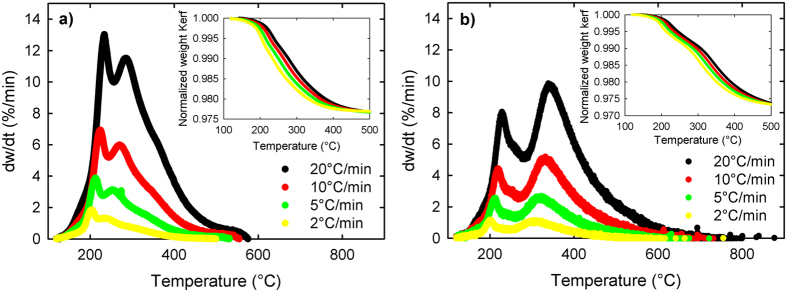
Rate of volatiles elimination for kerf at various heating rates (**a**) in air and (**b**) in N_2_. Insets show carbon elimination from kerf in the given atmosphere.

**Figure 5:**
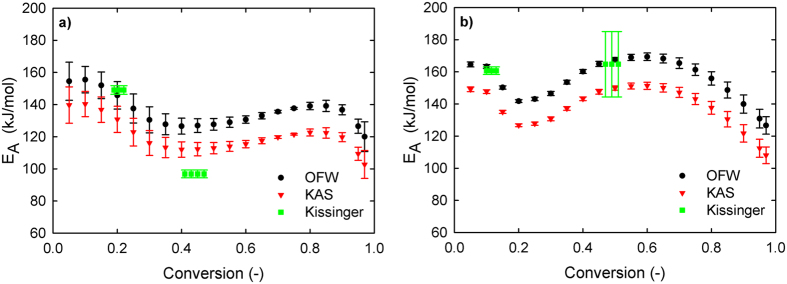
E_A_ for volatile elimination from kerf using OFW, KAS and Kissinger methods as a function of conversion (**a**) in air and (**b**) in N_2_. At low conversion both reactions display a similar behavior, but as reaction continues the N_2_ case displays higher E_A_ compared to air. Error bars represent the standard deviation.

Kinetics of carbon elimination in kerf were evaluated using three thermogravimetric analysis methods: OFW, KAS and Kissinger. The three methods are used in conjunction as their combined results provide more certainty on the accuracy of the obtained activation energy^22^. The raw quadruplet data at four different heating rates and its derivatives are shown for air in Fig. 4(a) and for N_2_ in Fig. 4(b). For information on determination of OFW and KAS E_A_ refer to the supplementary section. The E_A_ determined with the Kissinger method was obtained by computing the peak values of the derivatives from insets of Fig. 4(a) and (b). The apparent E_A_ determination results from the three methods are compared in Fig. 5(a) for air and Fig. 5(b) for N_2_. The OFW and KAS methods provide a similar plot as they are both isoconversional methods, yet the KAS determined E_A_ is considered to be a more accurate method^22^. The first peak for both gases shows a rather similar behavior even though in air the E_A_ is about 10 kJ/mol lower than in N_2_. However, the second peak for E_A_ is significantly more elevated in N_2_ than in air, suggesting a radically different mechanism for this second stage.

### Stages of the process

#### Carbon elimination in kerf is a two stage process

The rate of weight loss as a function of temperature exhibits two peaks, both in air, and in N_2_ (Fig. 4). The similar two-peak dependence is observed for the apparent activation energy as a function of conversion. Two peak phenomenon is the first fingerprint that our process is characterized by two stages. These stages are well distinguished by the products which are observed within the different temperature domains. (See Fig. 2 with the elemental characterization of corresponding volatiles).

GCMS for both air atmospheres and N_2_ shows two differentiated processes. In air, C_n_H_m_ compounds are observed up to 350 °C and not above this temperature. In the case of N_2_, saturated C_n_H_m_ compounds at temperatures above 450 °C, become less abundant, and unsaturated C_n_H_m_ compounds become more dominant. This comes in accordance with Arisawa *et al*.^23^, who reported that below this temperature, PEG forms both volatiles and a higher MW residue. Above this temperature, both PEG and its higher MW residue begin to decompose. Similar observations have been made for PEG decompositions at low pressure^28^. All of these facts provide evidence that carbon elimination in kerf is a two stage process.

### Apparent activation energy and stages of the process

Apparent activation energies, both for air and N_2_ atmospheres, as a function of conversion are not constant. Moreover, they are not constant even within the stages selected. This is consistent with observations of E_A_ behavior on PEG at comparable conditions by Arisawa *et al*.^23^. The two-peak apparent energy dependence is observed for the N_2_ atmosphere. Also the similar dependence is presented for the air atmosphere. For both cases, one can distinguish the clear ‘volcano-shape’ peculiarities of these dependences.

In the kinetic and catalytic literature, the observed increase in the apparent activation energy is typically attributed to the endothermic reactions, while the decrease is attributed to exothermic reactions^39^,^40^,^41^. The decrease in E_A_ between the two peaks for either air or N_2_ shows the point where the exothermic process (likely, polymerization) provides energy for volatiles to evolve but also diminishes the rate of total volatiles being released at that particular temperature. Then, in the second stage, the endothermic process (cleavage of molecules) produces the increase in E_A_ and releases more mass in the gas phase. Generally, two stages of our process present an interplay between the endothermic and exothermic factors.

All these facts provide evidence that our process is complex, displays at least two stages, and every stage is not an elementary reaction.

### Chemical reactions. Preliminary considerations

Evolution of chemical composition in the course of the TGA provides vast information about the complexity of our process within each stage.

#### First stage

Both atmospheres display a qualitatively analogous first stage, yet with different magnitudes. Based on GCMS (Fig. 2), oxygenated compounds, released as volatiles in the N_2_ atmosphere are abundant at the beginning of the 1^st^ peak (Fig. 4), but become scarce after 350 °C. On the other hand, in air for the same temperature domains, the oxygenated compounds ratio remains constant. This suggests that for the first stage, volatiles are evolving from comparably analogous mechanisms where the polymer cleaves itself using its own oxygen atoms to favor bond rupture. This holds true for both atmospheres (air and N_2_), although the magnitude of the effect is different. Moisture and low MW volatiles can be ruled out as the kerf sample was heated up to 120 °C for 1 hr and TGA showed a plateau in weight loss.

Furthermore, the results for the formation of C_n_H_m_ aliphatic compounds for pyrolysis (N_2_ atmosphere) conditions show firstly an increasing degree of polymerization with progressively increasing chain length. At temperatures below 350 °C, highly saturated linear chains form and volatize, and above that temperature more unsaturated compounds become prevalent. This is in agreement with Lattimer *et al*.^27^ who report that up to their tested temperature of 325 °C, in pyrolysis, no unsaturated compounds were found on volatilized products.

#### Second stage

In the second stage domain, a larger fraction of oxygenated compounds is found via GCMS in air rather than in N_2_. This indicates that in the air atmosphere, either oxygen reacts with the polymer and gets incorporated into the volatile chains as part of the reaction mechanism or the evolved gases oxidize in the gas phase. For N_2_, a relative lower abundance of oxygenated compounds indicates that as oxygen becomes less abundant in the remaining polymeric chain, C-C cleavage turns more dominant, yet not the sole reaction pathway. In the case of air atmosphere, C_n_H_m_ saturated compounds are not detected beyond 350 °C, and unsaturated C_n_H_m_ compounds were not found at any temperature.

### Higher MW polymer formation

Based on the FTIR results in N_2,_ as shown in Fig. 3(b), a small peak visible above 700 °C, is attributable to tar. Additionally, 12% of the original TC cannot be removed. This proves that pyrolysis is not enough for full carbon elimination of kerf lubricant. Lattimer *et al*.^27^ similarly observed a dark colored PEG residue attributable to highly unsaturated organics. In the case of N_2_, this higher MW PEG residue strongly influences the second peak decomposition features. Furthermore C-O and C-C bond cleavage (volatile formation) competes with radical recombination generating an even higher MW residue (tar), in agreement with previous studies^23^,^42^,^43^.

### Analysis of apparent E_A_ dependences

As indicated, the apparent EA dependence on temperature is characterized by two peaks similar to the rate of volatiles elimination. We considered it as a fingerprint of the two stage process. However even within one stage this apparent energy is not constant. Obviously it is the strong kinetic fingerprint that each stage is not an elementary reaction. Moreover, the maximum of the apparent energy for each stage can be interpreted as a result of the complicated interplay between at least two reactions, endothermic for decomposition reactions and exothermic for polymerization reactions. This interplay can be attributed both to the first and second stage.

### Detailed mechanism

**Figure 6:**
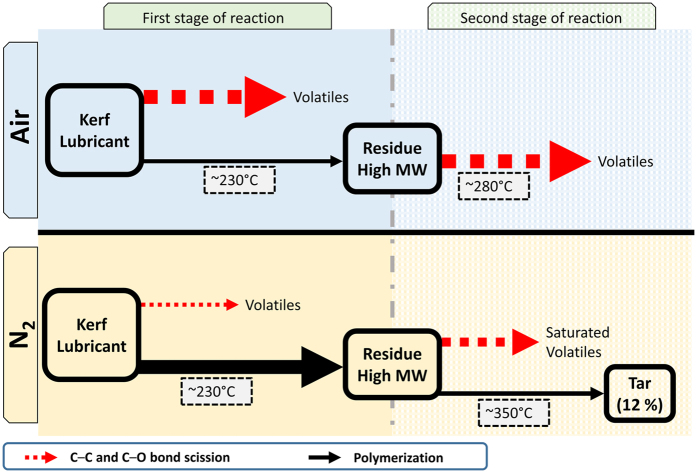
Two step mechanism for carbon elimination in kerf lubricant. Arrow thickness shows qualitative dominance for each parallel reaction pathway. At the end of the reaction in N_2_ 12% of carbon remains as tar.

Based on the presented composition and kinetic data and supported by the literature for PEG, a mechanism for the elimination of carbon from the slicing lubricant in kerf is presented graphically in Fig. 6.

#### In air

The reduction of mass during TGA in air can be explained by the formation of species with lower MW due to thermal degradation released to the gas phase upon formation. For the first stage, there are two competing types of reactions during thermal oxidation of PEG: oxidative degradation of PEG by a chain scission mechanism, and polymerization of PEG by loose crosslinking. The process of degradation consists primarily of competing C-C and C-O bond cleavage occurring in parallel at arguably comparable rates^28^. This is not different for the decomposition of polypropylene Glycol (PPG) or its related compounds^24^. During TGA in air, PEG undergoes thermal degradation forming lower MW species which are released into the gas phase, causing weight loss.

The second stage consists of the higher MW PEG undergoing full degradation into volatiles, with a peak in the formation of volatiles around 280 °C. No remaining carbon could be measured after exposing the sample up to 900 °C using a heating rate of 20 °C/min.

#### In N_2_

The proposed mechanism for kerf lubricant degradation in N_2_ involves also two types of reaction as in air, each comprising different mechanisms. In the first stage, the two overall processes occur in parallel. Competing C–C and C–O scissions take place to form volatiles, while at the same time, a higher MW polymer is formed at the same temperature as in the case of air (230 °C) hinting that the process might be the same in both atmospheres. Whereas in air the formation of volatiles dominates, in N_2_, the conditions favor the formation of high MW residue.

In the second stage in N_2_, the same two processes occur as in the first stage, C–O and C–C bond scissions compete to form volatiles. Those volatiles include a large fraction of saturated hydrocarbons in the gas phase that are not seen in the corresponding second stage in air. In N_2_, similar to the first stage, the polymerization continues and forms a tarry residue. This residue accounts for about 12% of the original TC content even after heating up to 900 °C using a heating rate of 20 °C/min. and shows that C elimination is not achievable under inert atmospheres. Tar was not observed in air.

### Interpretation of ‘volcano-shape’ energetic dependences

Our activation energy curves are similar to volcano-shape energetic dependences (volcano curves) which have been found in many heterogeneous chemical systems, particularly in gas-solid catalytic systems Typically, the interpretation of these interesting effects is presented based on linear correlations between the activation energy and the reaction enthalpy, Broensted-Evans-Polyani correlations, with the different sign, positive or negative, for endothermic and exothermic reactions, respectively^44^. In a simple way, the volcano curve can be explained by the compromise between endothermic and exothermic reactions.

In summary, we studied the carbon elimination from kerf and defined it as a two stage process based on TGA analysis. The activation energy for carbon elimination from kerf and its evolution was obtained in air and N_2_ atmosphere from room temperature to 900 °C. We developed a mechanistic model based on characterization tools including TC, FTIR, and GCMS and supported the model by the PEG decomposition literature. This mechanism is proposed as the skeleton of the actual reaction mechanism for the thermal decomposition of slicing lubricant in kerf in air and N_2_. In the first stage of our proposed model, the reaction rate is dominated by long hydrocarbon chains undergoing depolymerization by rupture of C-C and C–O bonds in competition with further polymerization. This happens in both atmospheres, but with different output ratios. The second stage is radically different for each atmosphere. In the case of air, this second stage is less energy demanding and the carbonaceous compounds polymerize to a lesser extent facilitating the removal of organics during the second stage. Complete carbon elimination in N_2_ atmosphere was not achieved under the temperatures and heating rates tested. The reaction rate is slower compared to air and yields a tarry byproduct (12% of original TC content) that cannot be easily eliminated by further heating.

We observe a higher rate of carbonaceous compound removal from kerf in air than in N_2_ atmosphere; however, this comes at the cost of oxidizing the silicon which is undesirable for kerf purification. The kinetics of carbon elimination should be linked with a model for silicon oxidation in kerf. A first approximation can be obtained from the Deal and Grove model^45^. However, the development of more accurate models for silicon oxidation from kerf is desirable. Further research on different concentrations of O_2_ and N_2_ could provide optimum conditions to maximize carbonaceous compound elimination while minimizing silicon oxidation. Such models will help to design processes to more effectively recycle and re-use kerf so as to bring down costs of Si wafers.

## Methods

### Sample characterization

Kerf was provided by SunEdison, Inc. (Saint Louis, MO) along with its elemental analysis determined by combustion infrared, inert gas fusion non dispersive infrared, and glow discharge mass spectrometry shown in Table 2. The surface area determination was performed using a Nova 200e BET instrument (Quantachrome Instruments, Boynton Beach, FL). Surface morphology and structural analysis were obtained by SEM in a FEI Nova 2300 SEM (FEI, Hillsboro, OR). Kerf was dried and its volatiles were removed by keeping the sample at 120 °C for 60 min before the experiments. Kerf was heated to various temperatures using TGA in a Q5000 IR thermogravimetric analyzer (TA Instruments, New Castle, DE) using a heating rate of 20 °C/min up to various temperatures at intervals of around 200 °C in either air (Airgas, St. Louis, MO) or ultra-high purity (99.999%) N_2_ (Airgas, St. Louis, MO). Samples were then quickly cooled down by shutting off the TGA furnace and characterized using TC determinations (Shimadzu SSM-5000, Schimadzu, Columbia, MD) with a solid sample module and FTIR spectroscopy (Nicolette Nexus 470 GMI, Ramsey, MN). The volatiles, evolved from kerf at different temperatures and atmospheres, were extracted from the TGA exhaust at the desired temperature using a gas tight 0.5 mL Luer syringe (Restek, Bellefonte, PA) and were immediately injected into a RTX 50 gas chromatography (GC) column equipped with mass spectroscopy (MS) (Agilent 5975 C Series GC/MSD, Santa Clara, CA). Peaks were integrated using Agilent Mass Hunter Quantitative Analysis Software and identified using the NIST library.

### TGA for kinetic analysis

TGA was used to determine the kinetics of carbon elimination in kerf. Each kerf sample was heated up to 120 °C and kept at that temperature for 60 min to remove water and volatiles from the sample. DEG as a component of the lubricant has a vapor pressure of 5.6 Torr at 120 °C^46^ and would be removed from the sample within this time (1 hr). Dried kerf samples were then subjected to four heating rates: 2, 5, 10 and 20 °C/min. under combustion (air) or pyrolysis (ultrapure N_2,_ 99.999%). The experiments were run in triplicate. A detailed discussion of the thermal analysis methods to determine E_A_ is provided in the supplementary section.

## Additional Information

**How to cite this article**: Vazquez-Pufleau, M. *et al*. Carbon elimination from silicon kerf: Thermogravimetric analysis and mechanistic considerations. *Sci. Rep.* **7**, 40535; doi: 10.1038/srep40535 (2017).

**Publisher's note:** Springer Nature remains neutral with regard to jurisdictional claims in published maps and institutional affiliations.

## Supplementary Material

Supplementary Information

## Figures and Tables

**Table 1:** Experimental plan for gaining kinetic information and mechanistic evidence on carbon elimination from kerf.

	Instrument	TGA Program	Sample	Information Obtained
1	TGA	Different heating rates (2, 5, 10 & 20 °C/min) up to 900 °C	Dried kerf	Activation energy (E_A_)
2	TC	20 °C/min up to desired temperature	TGA treated kerf	Total remaining carbon
3	GCMS	20 °C/min up to 900 °C	Kerf volatiles from TGA	Evolved gas composition
4	FTIR	20 °C/min up to desired temperature	Residue kerf from TGA	Species remaining in kerf
5	SEM	20 °C/min up to 900 °C	Kerf after TGA	Sample morphology

**Table 2:** Kerf elemental analysis provided by SunEdison, Inc.

Element	Composition %
Silicon	>84
Oxygen	13
Carbon	1.3
Aluminum	0.3
Nickel	0.015
Iron	0.0035
