# Supplementary material for: Carbon elimination from silicon kerf: Thermogravimetric analysis and mechanistic considerations
Source: Sci Rep. 2017 Jan 18;7:40535. doi: 10.1038/srep40535 (PMC5241785; doi:10.1038/srep40535)
Supplement: Supplementary Information [file srep40535-s1.pdf]

Supplementary information

Carbon elimination from silicon kerf: Thermogravimetric analysis and mechanistic considerations

Miguel Vazquez-Pufleau[1], Tandeep S. Chadha[1], Gregory Yablonsky[2], and Pratim Biswas[1,*]
[1] Aerosol and Air Quality Research Laboratory, Department of Energy, Environmental & Chemical Engineering, Washington University in St. Louis, St. Louis, MO 63130, USA
[2] Parks College, Department of Chemistry, Saint Louis University, St. Louis, MO 63103, USA

TGA vs TC conversion

TC is considered to be a less biased instrument to determine the total amount of carbon present in the sample. This becomes evident in the case of samples treated in air, where carbon is eliminated at a slightly lower rate than predicted by TGA, reaching full conversion with a small delay. In the case of samples treated in N_2, a similar delay in conversion is observed but full carbon removal is not achieved under the examined conditions (up to 900°C). Conversion is calculated using the classical definition (1- reactant current concentration/ reactant initial concentration). The discrepancy in the values between TGA and TC for the N_2 atmosphere is due to the different bases used for each. On one hand TGA conversion is based on the minimum of the weight measured. On the other hand TC conversion is based on the absolute value reported by the instrument.

Determination of EA

Most of the kinetic methods used in thermal analysis consider the reaction rate to be a function of only two variables[1]:

$$\frac{d\alpha}{dt} = k(T)f(\alpha) \quad (1)$$

, where k is the reaction rate constant, α the conversion, and T the temperature. Since isoconversional methods are based on the idea that the reaction rate at a constant conversion is only a function of temperature, we compared four sets of data using different heating rates, defined by:

$$\beta = \frac{dT}{dt} = \text{constant} \quad (2)$$

, where β is the heating rate, T is the temperature and t is time.

Since the Kissinger method uses only the peak rates providing single point values for E_A, we extend our analysis to include two isoconversional methods: the Kissinger-Akahira-Sunose (KAS) method and the Ozawa-Flynn-Wall (OFW) method used to obtain E_A as a function of temperature.

Kissinger Method

Under the condition of maximum reaction rate:

$$\frac{d^2\alpha}{dt^2} = \left[\frac{E\beta}{RT_m{}^2} + Af'(\alpha_m)e^{\left(-\frac{E_A}{RT_m}\right)}\right]\left(\frac{d\alpha}{dt}\right)_m = 0 \quad (3)$$

Rearranging

$$\ln\left(\frac{\beta}{T_m{}^2}\right) = \ln\left(-\frac{AR}{E_A}f'(\alpha_m)\right) - \frac{E_A}{RT_m} \quad (4)$$

, where β is the heating rate in °C/min, R is the gas constant (8.314J/molK) and E_A the activation energy (J/mol), m indicates values corresponding to maximum rate, The assumption for the Kissinger method[1] is that $f(\alpha)_m$ is independent of the heating rate so the term f´ is constant. This assumption is valid for n-th order kinetics and Avrami-Erofeev models[1].

Ozawa-Flynn-Wall Method

$$ln(\beta) = Const - 1.052\left(\frac{E_{A,\alpha}}{RT_\alpha}\right) \quad (5)$$

The value of the coefficient equal to 1.052 is known as the Doyle approximation[2].

Kissinger-Akahira-Sunose Method

$$ln\left(\frac{\beta}{T_\alpha^{1.92}}\right) = Const - 1.0008\left(\frac{E_{A,\alpha}}{RT_\alpha}\right) \quad (6)$$

The coefficient 1.0008 represents the Starink et al. approximation[3].

Figure S1 (a) and Figure S1 (b) show the OFW method plots for obtaining the activation energy. Similarly, for the KAS method Figure S2 (a) and Figure S2 (b) were used. The Kissinger method produces only one E_A per the given peak whereas isoconversional models provide E_A for all the range of reaction temperatures which make it more suitable. However, a combination of both gives more certainty in the accuracy of the E_A determination.

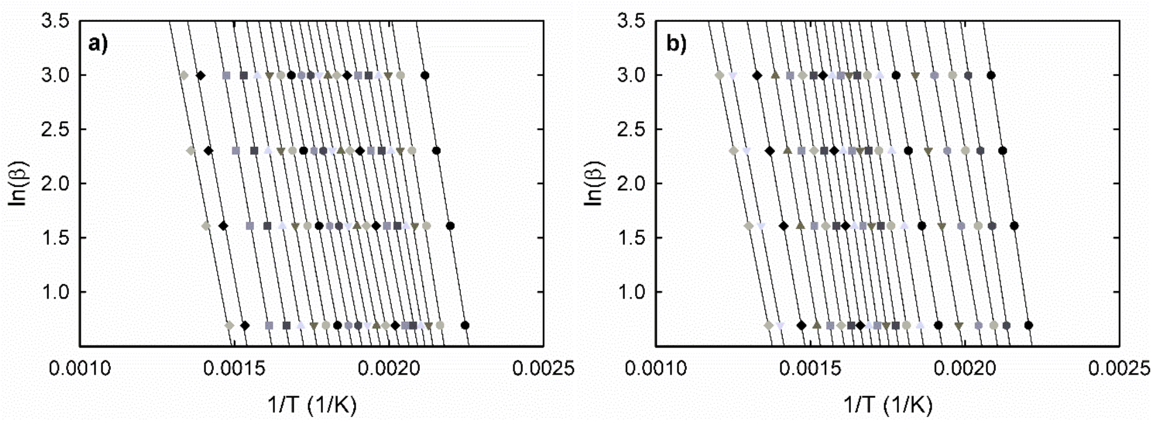

Figure S1. OFW method to calculate E_A (**a**) in air and (**b**) in N_2. High regression values are observed

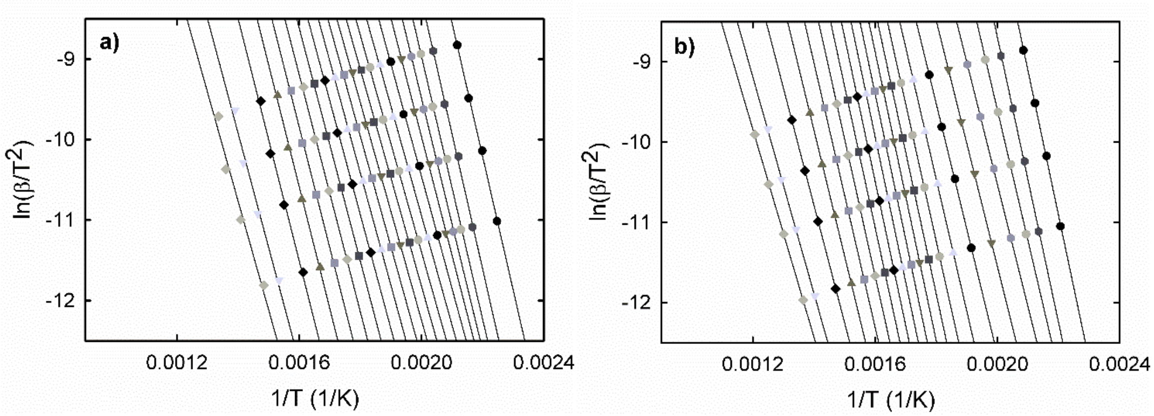

Figure S2. KAS method to calculate E_A (**a**) in air and (b) in N_2. The data quadruplet shows consistency and a high linear regression value indicating experimental data reliability.

Figure S3 compares the reaction rate with the values of the normalized rate by dividing it by the concentration and also dividing it by the concentration to the phenomenological power law model. The objective of this is to show the behavior of rate when the concentration effect is eliminated, as if the reaction rate proceeded without conversion. A sharp slope indicates exothermicity whereas a less steep or even a change in the slope sign is an indication of endothermicity.

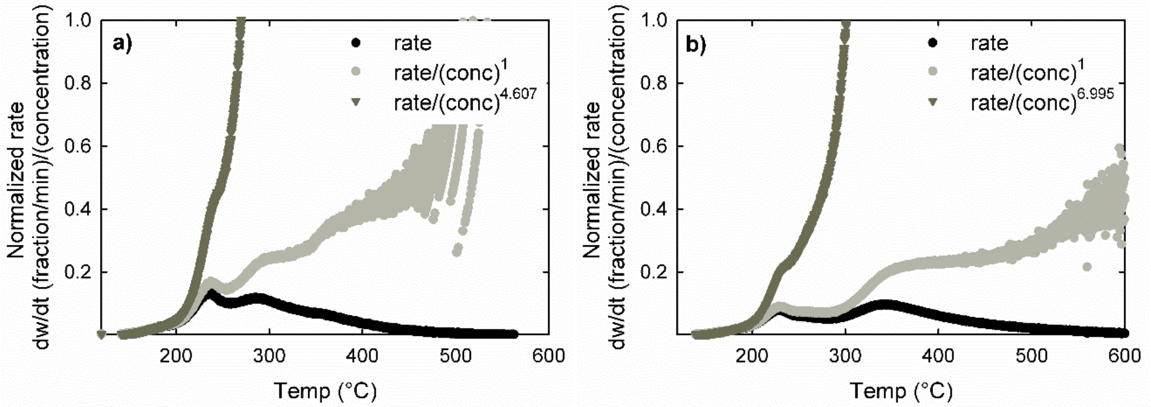

Figure S3. Normalized reaction rate in (**a**) air and (**b**) in N_2.The sharp slope indicates both reactions are predominantly exothermic.

PEG literature discussion

The degradation mechanism of PEG is complex. It decomposes endothermically under pyrolytic conditions according to Arisawa et al.[4] but exothermically in oxidative processes according to Lin et al.[5] In addition, even under oxygenic conditions, the degradation inside a thick sample would still be a pyrolysis process. Several authors have reported E_A ranging from 129 to 209 KJ/mol for the decomposition of PEG with MW from around 10^3 to 10^5 g/mol at temperatures of 315°C – 410°C as summarized by Arisawa et al.[6] The degradation temperature of PEG rises as the MW increases, but after about 1000 Da the maximum degradation temperature is rather constant at about 350°C according to Voorhees et al.[7] The primary degradation is above 325°C while the secondary is after 450°C. They also suggest that PEG undergoes a series of homolytic cleavages between the C-C and the C-O bonds followed by disproportionation and reactions of hydrogen abstraction. Arisawa et al.[4] report that C-O and C-C homolysis occur approximately at the same rate between 370 and 550°C. Saint de Claire et al.[8] suggest that the reaction mechanism initiates by hydroperoxide dissociation, propagates by alkoxy radicals and chain growth, and terminates when two radicals neutralize each other. However, this last step is heavily diffusion dependent.

According to Han et al. [9], one of the specific degradation mechanisms for PEG is the volatile formation from random chain scission phenomena, in which PEG reacts with oxygen to form a thermally sensitive peroxides that decompose via a radical mechanism to produce formic esters. Regarding the random chain scission phenomena, there seems to be dispute about which bond breaks at the initial stage of polymeric pyrolysis. Fares et al.[10] indicate that C-O homolysis dominates over C-C homolysis in the first stages of pyrolysis. Lattimer et al.[11] also report that at relatively low temperatures (150°C) homolytic cleavage of C-O bonds is the most important

degradation step. Arisawa et al.[4] on the other hand suggest that the rates of both cleavages are comparable.

Pielichowski et al.[12] found that PEG non oxidative decomposition yielded an apparent E_A of 145-180 kJ/mol, in agreement with our own results for N_2 decomposition (120-170 kJ/mol). Kitahara et al.[13] on the other hand reported E_A for three PEG pyrolysis products ranging from 155 to 214 kJ/mol and a reaction rate peak at 470°C instead of our observed 350°C. Kithara's higher E_A compared to ours can be explained by the MW of their PEG being 2 million atomic units, whereas ours is below a few thousand. The expected C-O bond strength is around 335 kJ/mol, much higher than the measured value. This is due to several reactions with differing E_A occurring in parallel. The complex reaction likely includes hydrogen abstraction reactions that display negative E_A[8]. As a result, the apparent E_A of the global reaction is significantly changed.

Volatiles characterization

Regarding the volatiles, the cyclized dioxolane products are formed from intramolecular radical recombination reactions where -O-C- radicals have a fundamental role[4]. Lattimer et al.[11] determined that the main PEG pyrolyzates are: dihydroxyl, methyl ether, vinyl ether, aldehyde, ethyl ether, methyl ether aldehyde, methyl vinyl ether, dialdehyde, and ethyl vinyl ether. According to Arisawa et al.[4] 2-methoxy-1,3-dioxolane forms from radicals produced by C-O homolysis of PEG and afterwards it undergoes intramolecular cyclization and finally complex radical recombination. This contributes to the higher E_A. On the other hand 1,3-dioxolane forms

by simple intramolecular cyclization of radical recombination after C-O homolysis of PEG displaying a lower E_A.

SEM characterization

To observe the effect of thermal treatment on morphology, SEM characterization was performed. Figure S4 (a) shows dried kerf, Figure S4 (b) displays kerf processed in air atmosphere at 900°C and Figure S4 (c) presents kerf after exposure in N_2 atmosphere at 900°C. No evident morphological changes are visible for the different processes. This supports the idea that the particle size does not change and introduce modifications into the silicon peaks.

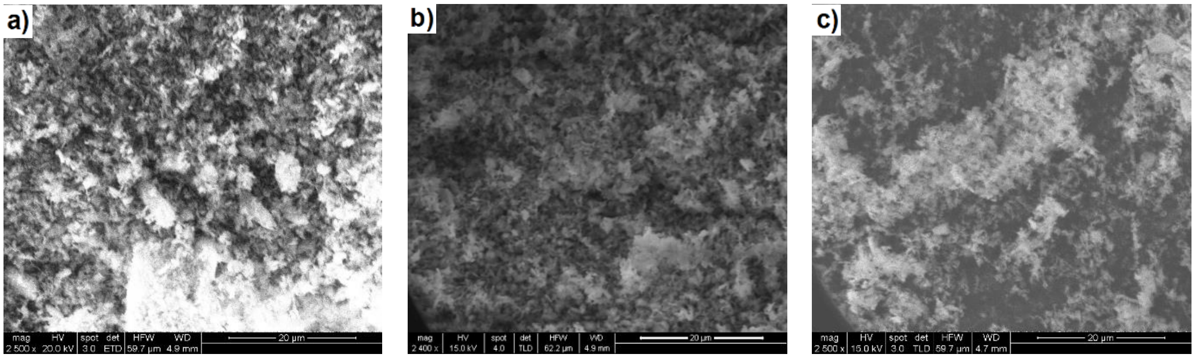

Figure S4. SEM of dried kerf at (**a**) RT, (**b**) heated to 900°C in air and (**c**) heated to 900°C in N_2. No significant differences are observed in the flake shape after thermal treatment.

XPS preliminary results

Nitrogenated compounds evolved as volatiles were observed to occur both in air and N_2. Preliminary results using XPS (not reported) show that the N_2 level decreases for a sample treated at a higher temperature suggesting that N_2 is part of the lubricant formulation and is located inside the polymeric chains rather than either adsorbed on the surface or extracted from the inert atmosphere or air. These nitrogenated compounds might be incorporated into the PEG as

antioxidants in the form of aromatic aminated additives since their properties against polymer free radical oxidative degradation[14] would extend the lubricant life span.

FTIR peak assignment

The assignments for peaks used in Figure 3 were based on the following table and references

Table S1. FTIR peak assignment

Molecular motion	Peaks FTIR	
	this study (cm^{-1})	Other studies (cm^{-1})
Si-O-Si bending vibration	805	800 [15]
CH_2 bending, rocking and twisting	960	964 [5,12]
Transverse optic (TO) SiO_2	1070	1074 [16]<br>1050 [17]<br>1060 [18]<br>1070 [19]
C-O stretching	1149	1149 Wang et al.<br>1153 [12]
Longitudinal optic (LO) SiO_2	1230	1235 [18]<br>1250 [19]<br>1230 [17]
CH_2 scissoring	1460	1470 [12]
Stretching and bending modes of aromatic hydrocarbon tar, soot or coke	1510	1500 [20]<br>1300-1600 [21]
Al-O vibration	1615	1612 [22]

Table S2 Group assignment based on GCMS for the air atmosphere

Air atmosphere				
**Retention time (min)**	**Relative abundance**	**Mass spectra data characteristic ion (70eV) m/z (%)**	**Tentative formula**	**Group assignment**
200°C				
4.911	53.45%	193(100), 209(41), 133(13), 194(11), 135(8), 179(3), 195(2), 44(2), 210(1), 151(1)	C6H18O3Si3	CnHmOlSik
6.943	8.54%	94(100), 67(69), 40(30), 39(11), 41(4), 44(3)	C4H4N2	CnHmNl
8.264	9.23%	42(100), 55(52), 39(40), 41(38), 98(29), 69(22), 70(6), 43(6), 40(2)	C17H28OSi	CnHmOlSik
8.508	2.51%	267(100), 268(20), 193(16), 251(12), 269(7), 126(5), 283(4)	C5H6N2	CnHmNl
9.904	7.24%	43(100), 44(11), 74(9), 45(8), 42(5), 41(2)	C5H6O2	CnHmOl
10.312	3.64%	105(100), 51(83), 77(73), 50(67), 106(67), 78(24), 74(16), 52(15), 39(6), 62(2)	C13H22O3Si2	CnHmOlSik
10.763	2.58%	42(100), 45(89), 43(66), 41(13), 44(12), 58(12), 39(6)	C3H6O2	CnHmOl
11.371	8.49%	55(100), 43(73), 41(66), 83(30), 70(28), 39(23), 69(21), 56(13), 77(12), 44(10)	C7H6O	CnHmOl
12.891	2.48%	207(100), 77(67), 45(21), 208(20), 209(12), 133(10), 191(7), 96(2), 59(1), 62(1)	C5H12O2	CnHmOl
14.939	1.84%	80(100), 53(68), 52(63), 51(40), 39(10), 79(7), 43(6), 50(5), 42(5), 40(4)	C6H10O	CnHmOl
230°C				
4.927	53.00%	193(100), 209(24), 133(11), 194(6), 208(4), 135(3)	C6H18O3Si3	CnHmOlSik
6.959	13.11%	0(9), 999(6), 591(4), 490(3), 198(5), 42(5), 52(5), 41(2), 44(2)	C4H4N2	CnHmNl
8.28	3.31%	45(100), 73(61), 267(39), 355(24), 43(16)	C8H7N3O4	CnHmNl
8.503	3.77%	42(100), 55(89), 41(51), 39(28), 98(26), 70(19), 69(19), 40(10), 43(9), 56(5)	C5H6N2	CnHmNl
9.57	1.67%	43(100), 45(18), 41(18), 74(14), 42(11), 44(7), 39(7), 67(3), 82(3), 58(3)	C10H30O5Si5	CnHmOlSik
9.904	12.54%	41(100), 57(74), 39(58), 43(46), 55(32), 44(20), 96(8), 73(8)	C6H10O	CnHmOl
10.769	4.24%	42(100), 45(71), 43(63), 41(36), 39(9), 44(8), 58(8)	C2H6N2O	CnHmNl
12.047	1.48%	41(100), 44(41), 43(22), 57(22), 55(9), 39(7), 42(7), 68(6), 81(6)	C6H10O3	CnHmOl
12.902	1.39%	0(4), 999(5), 705(4), 558(6), 447(5), 70(24), 83(23), 42(22), 56(22), 97(18)	C5H12O2	CnHmOl
13.512	1.20%	67(100), 39(67), 54(54), 41(45), 82(35), 207(32), 51(19), 50(18), 53(16), 79(4)	C9H14O4	CnHmOl
14.939	4.29%	207(100), 73(91), 43(76), 45(49), 44(9), 58(1), 42(1)	C8H18O	CnHmOl

300°C				
3.983	10.87%	207(100), 43(16), 77(14), 208(12), 41(5), 42(4), 39(3), 209(2), 45(2), 133(2)	C6H10	CnHm
4.455	1.41%	88(100), 43(82), 58(79), 42(40), 57(28), 44(20), 41(15), 39(1), 45(1)	C12H18O2Si	CnHmOlSik
4.974	3.05%	73(100), 45(48), 41(13), 42(12), 43(12), 39(7), 57(2)	C17H30OSi	CnHmOlSik
5.914	5.71%	41(100), 59(99), 87(78), 43(76), 57(55), 39(51), 42(30), 45(27), 58(23), 44(21)	C4H8O2	CnHmOl
6.232	1.93%	87(100), 59(73), 43(45), 41(35), 42(32), 57(30), 39(25), 72(18), 45(10), 44(8)	C5H10O2	CnHmOl
6.508	11.61%	43(100), 41(55), 52(52), 51(46), 39(44), 80(44), 42(40), 45(40), 53(35), 79(29)	C6H12O2	CnHmOl
6.731	6.29%	43(100), 39(47), 42(39), 114(26), 41(25), 45(12), 40(10), 44(5), 73(4), 55(4)	C6H12O2	CnHmOl
7.002	11.31%	42(100), 45(79), 43(72), 41(71), 39(30), 116(15), 40(5), 72(2), 101(1)	C8H12O2	CnHmOl
7.532	13.02%	41(100), 39(62), 43(48), 98(41), 55(29), 69(25), 51(7), 42(6)	C4H10	CnHm
7.718	2.40%	43(100), 94(60), 41(41), 39(40), 40(37), 67(33), 42(30), 45(24), 53(3), 52(3)	C4H8O2	CnHmOl
7.941	0.76%	41(100), 55(64), 43(44), 69(38), 39(26), 56(25), 44(13), 93(8), 70(7), 63(6)	C6H10O	CnHmOl
8.588	7.02%	43(100), 45(22), 39(12), 74(11), 53(3), 42(3)	C5H6N2	CnHmNl
9.089	2.19%	43(100), 42(10), 86(5), 67(4), 41(3), 44(3), 116(3)	C7H14	CnHm
9.373	0.71%	42(100), 55(76), 39(58), 41(57), 43(15), 98(15), 40(14), 70(14), 69(13), 53(6)	C3H6O3	CnHmOl
9.793	2.54%	43(100), 45(19), 41(18), 42(12), 74(9), 58(6), 39(6), 44(5), 69(2), 97(2)	C4H6O2	CnHmOl
9.916	2.98%	42(100), 41(74), 39(20), 40(18), 86(9), 56(8), 44(6), 43(5), 55(4), 53(2)	C5H10	CnHm
10.797	2.25%	41(100), 43(44), 55(36), 39(29), 57(24), 44(22), 42(15), 67(6), 53(5)	C3HnO3	CnHmOl
11.769	6.29%	42(100), 43(52), 41(29), 45(28), 39(23), 44(14), 40(2)	C4H6O2	CnHmOl
12.048	0.73%	41(100), 43(59), 57(46), 55(40), 39(31), 44(27), 67(16), 56(12), 70(10), 42(10)	C6H10O3	CnHmOl
12.859	1.38%	41(100), 43(62), 55(50), 69(45), 56(41), 70(33), 57(29), 42(23), 83(21), 68(17)	C4H6O2	CnHmOl
13.512	1.29%	54(100), 39(97), 67(90), 41(65), 82(55), 51(54), 50(28), 207(27), 53(17), 79(15)	C12H24	CnHm
14.939	4.24%	41(100), 44(22), 39(16), 43(15), 42(3)	C10H20	CnHm
350°C				
4.067	27.82%	88(100), 43(58), 58(51), 44(24), 42(18), 57(10), 41(1)	C6H10	CnHm

5.261	4.02%	52(100), 51(81), 79(77), 50(57), 80(53), 53(24), 39(22), 43(12), 40(10), 41(9)	C3H8	CnHm
5.908	5.97%	43(100), 42(33), 41(25), 281(21), 39(21), 114(12), 45(3), 40(1), 44(1), 282(1)	C3H4O3	CnHmOl
6.938	18.71%	41(100), 55(40), 39(26), 67(22), 94(19), 43(17), 56(16), 42(15), 69(12), 40(9)	C5H5N	CnHmNl
7.521	7.04%	41(100), 55(83), 43(37), 39(9), 56(7), 140(6), 44(6)	C4H10	CnHm
8.588	5.42%	93(100), 39(65), 41(44), 66(28), 55(27), 92(18), 65(10), 67(8), 63(7), 43(7)	C7H12O	CnHmOl
8.901	1.16%	39(100), 43(50), 53(47), 82(46), 54(25)	C12H20O4	CnHmOl
9.103	3.22%	43(100), 67(27), 39(6)	C6H7N	CnHmNl
9.373	0.66%	55(100), 42(87), 39(54), 41(49), 98(28), 70(6), 43(5), 40(5)	C5H6O2	CnHmOl
9.782	0.68%	75(100), 103(73), 133(71), 43(31), 45(27), 59(24), 61(22), 117(19), 77(13), 44(6)	C8H14O2	CnHmOl
9.92	1.93%	68(100), 39(43), 40(16), 96(6), 42(5)	C5H10	CnHm
10.811	2.61%	42(100), 41(75), 39(29), 40(19), 56(15), 86(15), 44(9), 55(4), 43(4), 53(3)	C6H16O2Si	CnHmOlSik
11.039	0.96%	42(100), 44(82), 43(75), 45(23), 41(20), 102(11), 58(8)	C4H4O	CnHmOl
11.745	13.98%	42(100), 41(41), 44(36), 43(21), 39(9), 45(3)	C4H6O2	CnHmOl
12.583	1.16%	41(100), 44(53), 43(52), 55(39), 39(23), 57(19), 42(9)	C4H10N2O	CnHmNl
12.865	1.59%	41(100), 55(58), 43(53), 70(31), 42(29), 39(29), 57(26), 56(25), 69(20), 44(14)	C4H10O2	CnHmOl
13.501	1.10%	79(100), 52(68), 50(50), 51(41), 281(29), 44(18), 80(6), 53(5), 39(5)	C4H8O2	CnHmOl
14.939	1.97%	117(100), 59(30), 89(24), 75(21), 44(17), 103(12), 45(4), 43(4), 101(3)	C7H16O	CnHmOl
450°C				
6.928	30.47%	75(100), 103(68), 133(54), 43(39), 45(34), 61(33), 77(33), 117(31), 59(24), 44(18)	C5H5N	CnHmNl
10.424	12.33%	42(100), 41(75), 44(16), 39(13), 56(6), 86(4), 40(2)	C5H12O2Si	CnHmOlSik
10.817	29.52%	103(100), 76(47), 44(47), 50(29), 43(27), 51(17), 61(10), 75(4), 39(3), 40(3)	C6H16O2Si	CnHmOlSik
11.756	21.57%	117(100), 89(58), 75(43), 59(39), 101(28), 103(27), 77(26), 45(25)	C4H6O2	CnHmOl
11.991	6.10%	103(100), 75(96), 133(61), 45(42), 43(41), 117(36), 77(33), 61(33), 44(26), 59(19)	C5H6N2	CnHmNl
550°C				
10.419	13.84%	77(100), 105(86), 51(77), 106(76), 50(56), 78(18), 52(5), 39(4), 74(4)	C8H20OSi	CnHmOlSik
10.811	32.71%	103(100), 43(63), 44(56), 50(42), 76(41), 61(37), 51(17)	C6H16O2Si	CnHmOlSik

11.374	18.78%	103(100), 43(63), 44(56), 50(42), 76(41), 61(37), 51(17)	C7H6O	CnHmOl
12.008	34.67%	103(100), 43(63), 44(56), 50(42), 76(41), 61(37), 51(17)	C7H5N	CnHmNl

Table S3 Group assignment based on GCMS for N_2 atmosphere

**Nitrogen atmosphere**				
**Retention time (min)**	**Relative abundance**	**Mass spectra data characteristic ion (70eV) m/z (%)**	**Tentative formula**	**Group assignment**
200 °C				
6.986	14.32%	51(100), 41(89), 39(87), 52(77), 53(67), 50(60), 44(58), 79(51), 40(42), 56(40)	C6H10	CnHm
7.474	9.28%	281(100), 43(31), 282(25), 73(22), 193(20), 44(20), 283(14), 192(14), 42(10), 56(8)	C8H24O4Si4	CnHmOlSik
8.211	7.11%	56(100), 43(59), 42(31), 41(29), 39(22), 44(14), 50(9), 51(7), 77(5), 99(3)	C4H10O	CnHmOl
8.577	6.98%	39(100), 41(90), 55(78), 40(59), 82(51), 56(49), 94(48), 67(44), 44(43), 42(42)	C6H12O	CnHmOl
9.108	2.83%	44(100), 39(88), 70(68), 66(26), 56(25), 57(18), 65(18), 41(15), 355(15), 73(14)	C5H13N	CnHmNl
9.57	4.41%	73(100), 267(42), 355(17), 44(13), 43(11), 268(8), 77(4), 266(4), 45(3), 78(2)	C10H30O5Si5	CnHmOlSik
9.904	6.10%	42(100), 39(93), 41(69), 55(50), 40(38), 70(37), 44(33), 69(21), 43(20), 98(18)	C6H10O	CnHmOl
11.374	3.81%	77(100), 106(73), 50(69), 44(61), 51(45), 52(36), 73(28), 39(27), 105(25), 78(14)	C7H6O	CnHmOl
11.75	12.46%	73(100), 41(92), 341(53), 42(45), 44(37), 93(37), 39(30), 40(26), 325(19), 429(16)	C12H36O6Si6	CnHmOlSik
12.461	6.82%	41(100), 40(39), 68(31), 39(27), 42(21), 44(17), 55(17), 83(8), 69(6), 58(5)	C4H5N3O2	CnHmNl
12.886	2.64%	43(100), 41(87), 44(83), 42(72), 45(50), 98(27), 39(23), 57(16), 58(16), 51(14)	C4H8O	CnHmOl
13.337	7.13%	68(100), 55(66), 39(54), 42(32), 44(25), 43(22), 126(21), 83(20), 41(19), 40(14)	C5H6N2O2	CnHmNl
13.926	3.99%	73(100), 44(33), 281(28), 95(21), 41(21), 40(15), 147(14), 74(14), 42(13), 54(12)	C14H27NOSi2	CnHmOlSik
14.854	12.10%	68(100), 39(69), 98(41), 53(38), 41(37), 44(33), 40(30), 42(24), 43(22), 96(20)	C6H6O3	CnHmOl
230°C				
6.948	30.08%	39(100), 42(67), 41(53), 45(50), 43(34), 40(23), 44(22), 52(22), 51(16), 50(11)	C6H8N2	CnHmNl
8.508	15.06%	94(100), 67(62), 40(56), 39(47), 52(28), 41(26), 42(22), 44(18), 51(17), 53(13)	C5H6N2	CnHmNl

9.909	38.11%	42(100), 39(70), 55(60), 40(26), 98(24), 41(17), 108(15), 107(14), 52(14), 70(13)	C6H10O	CnHmOl
11.373	9.83%	105(100), 50(98), 51(96), 77(87), 106(52), 52(40), 39(35), 74(35), 63(35), 78(24)	C7H6O	CnHmOl
11.766	6.92%	42(100), 41(87), 39(63), 40(55), 44(50), 51(7), 45(7), 55(7), 86(7), 73(7)	C3H6	CnHm
300°C				
4.014	27.00%	39(100), 67(97), 54(67), 41(62), 51(55), 82(31), 53(26), 50(22), 52(15), 65(14)	C6H10	CnHm
5.871	5.72%	88(100), 43(56), 58(54), 42(45), 57(38), 44(30), 41(9), 45(4), 87(1), 65(1)	C4H8O2	CnHmOl
6.535	9.22%	41(100), 87(97), 59(81), 42(70), 43(66), 39(43), 44(22), 58(21), 72(18), 45(18)	C6H12O2	CnHmOl
7.007	11.70%	42(100), 45(50), 39(48), 43(47), 51(41), 52(40), 41(38), 50(32), 53(22), 40(21)	C4H8N2O2	CnHmNl
7.532	2.69%	43(100), 42(61), 39(46), 44(39), 41(30), 281(27), 45(13), 114(10), 40(7), 193(7)	C3H8	CnHm
7.691	5.12%	42(100), 41(60), 45(42), 39(35), 43(32), 40(18), 116(10), 44(9), 57(6), 72(6)	C4H8O	CnHmOl
8.195	2.99%	56(100), 43(50), 41(22), 42(19), 39(8), 44(6), 99(4), 87(2), 52(2), 51(2)	C6H13N	CnHmNl
8.583	7.99%	41(100), 55(36), 39(32), 56(20), 43(17), 57(15), 70(13), 42(12), 69(10), 53(9)	C8H16	CnHm
8.896	2.38%	41(100), 55(71), 43(57), 39(51), 44(49), 42(24), 56(17), 54(11), 115(8), 45(7)	C7H12O	CnHmOl
9.092	1.64%	41(100), 55(79), 39(62), 43(38), 93(28), 44(27), 42(24), 51(24), 69(16), 70(14)	C6H8O2	CnHmOl
9.909	9.54%	42(100), 55(65), 39(52), 41(35), 40(24), 98(21), 107(20), 70(15), 51(15), 43(14)	C6H10O	CnHmOl
10.328	2.32%	41(100), 42(91), 44(78), 82(59), 55(35), 40(33), 96(28), 43(24), 39(23), 51(19)	C6H11N	CnHmNl
11.363	2.33%	77(100), 50(91), 106(46), 39(37), 51(24), 105(23), 40(19), 44(18), 52(8), 78(8)	C7H6O	CnHmOl
11.761	4.86%	41(100), 42(66), 39(38), 44(20), 40(14), 56(10), 53(9), 55(7), 45(4), 43(3)	C4H6O2	CnHmOl
12.047	2.23%	41(100), 43(66), 39(59), 56(35), 44(31), 42(30), 57(27), 40(16), 55(15), 69(15)	C9H18O	CnHmOl
13.507	1.17%	41(100), 43(52), 44(50), 55(34), 67(29), 42(22), 39(21), 56(21), 95(18), 69(12)	C8H16O	CnHmOl
15.422	1.10%	42(100), 41(65), 55(65), 39(32), 40(24), 44(17), 51(7), 56(7), 52(4), 91(3)	C5H10	CnHm
350°C				
5.866	8.83%	88(100), 43(82), 58(74), 42(47), 57(37), 44(25), 45(13), 41(8), 87(6), 39(4)	C4H8O2	CnHmOl
6.222	2.68%	73(100), 41(44), 45(31), 39(28), 57(18), 42(17), 55(12), 40(12), 43(10), 72(6)	C5H10O2	CnHmOl

6.529	9.39%	43(100), 41(95), 87(60), 42(57), 39(54), 59(53), 57(41), 45(21), 44(18), 55(17)	C6H12O2	CnHmOl
6.715	15.03%	87(100), 59(72), 41(69), 43(45), 42(43), 57(35), 39(33), 72(13), 45(12), 55(11)	c6H12O2	CnHmOl
7.537	1.93%	43(100), 39(37), 41(36), 42(32), 40(12), 114(10), 45(6), 85(4), 57(3), 56(3)	C4H10	CnHm
7.697	2.39%	42(100), 41(65), 43(60), 45(42), 39(37), 40(17), 116(13), 72(11), 101(11), 57(8)	C6H12O2	CnHmOl
7.93	4.58%	41(100), 39(83), 98(40), 55(40), 50(30), 53(30), 51(25), 66(21), 52(16), 69(16)	C6H10O	CnHmOl
8.381	1.90%	41(100), 43(68), 42(31), 39(28), 57(27), 69(20), 71(20), 55(18), 56(17), 98(16)	C10H22	CnHm
8.588	19.85%	41(100), 39(61), 55(43), 43(35), 56(26), 70(21), 57(19), 42(19), 69(18), 53(15)	C10H20	CnHm
8.89	8.19%	41(100), 55(69), 43(44), 39(42), 56(26), 69(22), 67(19), 53(19), 42(16), 54(16)	C10H20	CnHm
9.087	7.62%	41(100), 55(62), 39(60), 43(40), 56(35), 70(28), 69(24), 53(21), 42(21), 67(20)	C10H20	CnHm
9.914	4.66%	42(100), 39(58), 41(41), 55(29), 40(17), 98(16), 69(14), 56(12), 51(11), 70(10)	C6H10O	CnHmOl
10.323	2.21%	107(100), 108(56), 52(49), 51(45), 39(41), 80(29), 53(22), 40(21), 50(17), 42(15)	C6H8N2	CnHmNl
11.469	2.92%	94(100), 42(70), 121(65), 39(63), 52(56), 122(44), 51(40), 53(39), 41(38), 50(23)	C7H10N2	CnHmNl
11.75	4.32%	41(100), 42(97), 39(61), 40(26), 93(13), 44(12), 43(11), 56(10), 53(10), 55(9)	C4H6O2	CnHmOl
13.512	0.53%	41(100), 43(66), 42(35), 55(33), 39(30), 44(25), 56(23), 77(23), 40(21), 57(17)	C10H20O	CnHmOl
13.995	0.94%	41(100), 43(78), 57(45), 55(34), 39(27), 44(26), 42(19), 56(16), 69(11), 71(11)	C8H18O	CnHmOl
14.616	0.46%	41(100), 55(76), 43(44), 69(32), 42(28), 111(27), 83(25), 51(19), 54(18), 57(18)	C10H20	CnHm
15.077	0.97%	106(100), 77(30), 39(22), 41(18), 135(18), 65(12), 79(12), 51(11), 43(10), 315(9)	C9H13N	CnHmNl
15.427	0.60%	42(100), 41(64), 55(50), 56(25), 393(24), 43(17), 40(16), 114(15), 44(13), 122(12)	C6H10O2	CnHmOl
450°C				
4.852	11.52%	207(100), 208(21), 191(13), 39(13), 41(9), 209(9), 103(7), 96(7), 133(6), 43(6)	C6H18O3Si3	CnHmOlSik
5.935	2.58%	88(100), 43(65), 57(34), 58(33), 42(30), 41(29), 44(29), 45(17), 39(15), 51(11)	C4H8O2	CnHmOl
6.264	2.59%	91(100), 92(72), 39(48), 63(22), 65(21), 51(21), 50(13), 41(9), 40(6), 62(6)	C7H8	Unsat. HC
6.916	14.98%	51(100), 52(83), 50(59), 79(49), 80(49), 53(41), 39(34), 49(19), 40(15), 281(14)	C6H8	Unsat. HC
7.468	4.83%	281(100), 193(43), 282(29), 73(24), 283(14), 43(13), 39(11), 191(9), 207(8), 280(8)	C8H24O4Si4	CnHmOlSik

8.004	6.77%	39(100), 93(94), 51(92), 66(88), 91(86), 50(83), 63(60), 92(50), 40(45), 65(42)	C6H7N	CnHmNl
8.588	12.08%	41(100), 55(51), 39(49), 43(34), 56(27), 42(22), 57(17), 69(16), 70(14), 83(11)	C10H20	CnHm
9.092	7.41%	41(100), 39(67), 55(47), 93(45), 43(42), 56(32), 67(32), 65(30), 40(25), 63(23)	C8H14O	CnHmOl
9.575	8.01%	73(100), 267(55), 355(24), 39(14), 356(13), 357(10), 268(9), 53(7), 251(7), 106(6)	C10H30O5Si5	CnHmOlSik
9.917	0.79%	39(100), 42(89), 55(72), 41(36), 98(18), 51(16), 40(12), 43(12), 50(11), 53(10)	C5H6O2	CnHmOl
10.026	2.87%	107(100), 108(55), 51(51), 39(49), 80(37), 106(36), 79(34), 52(34), 53(23), 77(20)	C7H9N	CnHmNl
10.307	5.95%	53(100), 39(69), 96(57), 67(53), 51(43), 42(37), 41(36), 40(29), 80(25), 50(23)	C6H8O	CnHmOl
10.747	1.25%	107(100), 65(99), 92(98), 51(91), 50(74), 39(60), 44(42), 106(40), 52(38), 78(35)	C7H9N	CnHmNl
11.851	16.23%	73(100), 341(31), 42(27), 93(20), 39(19), 41(18), 429(15), 325(13), 74(9), 66(8)	C12H36O6Si6	CnHmOlSik
13.941	2.15%	73(100), 281(18), 41(18), 327(17), 147(15), 44(10), 39(7), 43(7), 40(6), 415(5)	C19H54O7Si7	CnHmOlSik
550°C				
3.059	7.74%	41(100), 40(77), 39(35), 52(6), 44(6), 42(5), 51(4), 53(4), 132(1)	C2H3N	CnHmNl
4.041	3.25%	207(100), 78(98), 54(89), 50(77), 52(66), 51(62), 44(60), 39(55), 40(53), 41(17)	C24H36O2Si2	CnHmOlSik
4.322	9.91%	78(100), 50(33), 51(28), 52(24), 207(18), 39(18), 63(11), 77(8), 44(6), 54(6)	C6H6	Unsat. HC
4.757	5.83%	207(100), 208(29), 133(18), 44(8), 191(5), 96(5), 209(4), 43(4), 40(2), 177(2)	C6H18O3Si3	CnHmOlSik
6.258	8.83%	91(100), 92(53), 63(53), 39(33), 51(32), 65(26), 50(16), 62(14), 52(9), 40(7)	C7H8	Unsat. HC
6.943	7.38%	52(100), 51(49), 79(49), 50(41), 80(20), 53(18), 40(18), 39(17), 49(8), 44(6)	C5H5N	CnHmNl
7.452	2.40%	281(100), 193(30), 73(28), 282(22), 44(14), 283(11), 43(7), 194(6), 39(5), 191(3)	C8H24O4Si4	CnHmOlSik
8.015	5.36%	91(100), 51(49), 93(40), 39(38), 78(31), 50(29), 66(19), 106(19), 77(18), 92(14)	C8H10	Unsat. HC
8.593	5.21%	41(100), 43(30), 55(23), 56(23), 69(19), 39(17), 57(17), 53(14), 70(14), 83(13)	C8H10	Unsat. HC
8.757	5.14%	80(100), 39(97), 81(86), 51(78), 41(74), 52(68), 50(58), 91(58), 53(47), 56(29)	C5H7N	CnHmNl
9.092	4.31%	78(100), 104(91), 51(86), 50(58), 77(44), 39(38), 63(35), 44(34), 102(26), 103(21)	C8H8	Unsat. HC
9.575	2.02%	73(100), 267(34), 355(18), 251(12), 40(11), 74(11), 59(9), 44(7), 78(6), 95(6)	C10H30O5Si5	CnHmOlSik
10.058	1.23%	107(100), 44(86), 39(78), 106(54), 52(52), 51(50), 40(37), 50(30), 108(26), 54(16)	C7H9N	CnHmNl

10.424	1.68%	39(100), 51(60), 94(47), 107(41), 44(37), 106(34), 120(28), 40(27), 80(26), 53(24)	C6H8N2	CnHmNl
10.763	1.25%	107(100), 50(96), 77(71), 51(69), 92(45), 44(43), 106(43), 39(42), 52(39), 117(39)	C7H9N	CnHmNl
11.368	1.65%	50(100), 77(64), 105(63), 51(58), 44(48), 106(46), 52(39), 91(22), 39(20), 49(17)	C7H6O	CnHmOl
11.803	10.63%	41(100), 93(93), 39(85), 66(72), 73(65), 42(63), 65(39), 44(34), 40(32), 341(24)	C6H7N	CnHmNl
12.201	1.53%	115(100), 116(37), 63(23), 89(21), 50(19), 117(18), 114(14), 51(13), 39(10), 44(9)	C9H8	Unsat. HC
13.236	3.98%	107(100), 106(98), 52(53), 77(52), 44(47), 51(46), 63(37), 50(35), 53(33), 41(32)	C7H9N	CnHmNl
13.995	4.81%	107(100), 106(92), 52(53), 77(52), 44(47), 51(41), 63(37), 50(35), 53(33), 41(32)	C9H18O	CnHmOl
14.621	1.58%	41(100), 55(62), 39(51), 44(34), 43(18), 83(18), 53(14), 70(13), 51(10), 118(10)	C6H10O	CnHmOl
15.072	1.80%	106(100), 77(28), 44(21), 41(20), 51(17), 39(12), 135(9), 65(6), 78(4), 40(4)	C9H13N	CnHmNl
16.265	2.47%	129(100), 69(79), 135(57), 58(54), 44(43), 51(43), 65(38), 63(33), 315(29), 75(25)	C5H7N	CnHmNl

References:

1 Vyazovkin, S. *et al.* ICTAC Kinetics Committee recommendations for performing kinetic computations on thermal analysis data. *Thermochim. Acta* **520,** 1-19, (2011).

2 Doyle, C. D. Estimating isothermal life from thermogravimetric data. *J. Appl. Polym. Sci.* **6,** 639-642, (1962).

3 Starink, M. J. The determination of activation energy from linear heating rate experiments: a comparison of the accuracy of isoconversion methods. *Thermochim. Acta* **404,** 163-176, (2003).

4 Arisawa, H. & Brill, T. B. Flash pyrolysis of polyethyleneglycol .1. Chemometric resolution of FTIR spectra of the volatile products at 370-550 degrees C. *Combust. Flame* **109,** 87-104, (1997).

5 Lin, Z., Han, X., Wang, T. & Li, S. Effects of adding nano metal powders on thermooxidative degradation of poly(ethylene glycol). *J. Therm. Anal. Calorim.* **91,** 709-714, (2008).

6 Arisawa, H. & Brill, T. B. Flash pyrolysis of polyethyleneglycol II: Kinetics determined by T-jump/FTIR spectroscopy. *Combust. Flame* **109,** 105-112, (1997).

7 Voorhees, K. J., Baugh, S. F. & Stevenson, D. N. An investigation of the thermal degradation of poly(ethylene glycol). *J. Anal. Appl. Pyrolysis* **30,** 47-57, (1994).

8 de Sainte Claire, P. Degradation of PEO in the solid state: A theoretical kinetic model. *Macromolecules* **42,** 3469-3482, (2009).

9 Han, S., Kim, C. & Kwon, D. Thermal/oxidative degradation and stabilization of polyethylene glycol. *Polymer* **38,** 317-323, (1997).

10 Fares, M. M., Hacaloglu, J. & Suzer, S. Characterization of degradation products of polyethylene oxide by pyrolysis mass spectrometry. *Eur. Polym. J.* **30,** 845-850, (1994).

11 P Lattimer, R. Mass spectral analysis of low-temperature pyrolysis products from poly (ethylene glycol). *J. Anal. Appl. Pyrolysis* **56,** 61-78, (2000).

12 Pielichowski, K. & Flejtuch, K. Non-oxidative thermal degradation of poly(ethylene oxide): kinetic and thermoanalytical study. *J. Anal. Appl. Pyrolysis* **73,** 131-138, (2005).

13 Kitahara, Y., Takahashi, S. & Fujii, T. Thermal analysis of polyethylene glycol: Evolved gas analysis with ion attachment mass spectrometry. *Chemosphere* **88,** 663-669, (2012).

14 Pospisil, J. Aromatic and heterocyclic amines in polymer stabilization. *Polysoaps/Stabilizers/Nitrogen-15 Nmr* **124,** 87-189, (1995).

15 Mansour, N., Momeni, A., Karimzadeh, R. & Amini, M. Surface effects on the luminescence properties of colloidal silicon nanocrystals in water. *Phys. Scr.* **87,** 035701, (2013).

16 Martinet, C. & Devine, R. A. B. Analysis of the vibrational mode spectra of amorphous SiO2 films. *J. Appl. Phys.* **77,** 4343-4348, (1995).

17 Asuha, H. K., Maida, O., Takahashi, M. & Iwasa, H. Nitric acid oxidation of Si to form ultrathin silicon dioxide layers with a low leakage current density. *J. Appl. Phys.* **94,** 7328-7335, (2003).

18 Queeney, K. T. *et al.* Infrared spectroscopic analysis of the Si/SiO2 interface structure of thermally oxidized silicon. *J. Appl. Phys.* **87,** 1322-1330, (2000).

19 Devine, R. A. B. Structural nature of the Si/SiO2 interface through infrared spectroscopy. *Appl. Phys. Lett.* **68,** 3108-3110, (1996).

20 Sun, M. *et al.* GC-MS and TG-FTIR study of petroleum ether extract and residue from low temperature coal tar. *Energy Fuels* **25,** 1140-1145, (2011).

21 Maroni, V. A. & Epperson, S. J. An in situ infrared spectroscopic investigation of the pyrolysis of ethylene glycol encapsulated in silica sodalite. *Vib. Spectrosc.* **27,** 43-51, (2001).

22 Zaki, M. I., Hasan, M. A., Al-Sagheer, F. A. & Pasupulety, L. In situ FTIR spectra of pyridine adsorbed on SiO_2–Al_2O_3, TiO_2, ZrO_2 and CeO_2: general considerations for the identification of acid sites on surfaces of finely divided metal oxides. *Colloids Surf., A* **190,** 261-274, (2001).

Table of content

List of Supplementary Tables

Table S1. FTIR peak assignment

Table S2 Group assignment based on GCMS for the air atmosphere

Table S3 Group assignment based on GCMS for the N_2 atmosphere

List of Supplementary Figures

Figure S1. OFW method to calculate E_A (**a**) in air and (**b**) in N_2. High regression values are observed

Figure S2. KAS method to calculate E_A (**a**) in air and (b) in N_2. The data quadruplet shows consistency and a high linear regression value indicating experimental data reliability.

Figure S3. Normalized reaction rate in (**a**) air and (**b**) in N_2.The sharp slope indicates both reactions are predominantly exothermic.

Figure S4. SEM of dried kerf at (**a**) RT, (**b**) heated to 900°C in air and (**c**) heated to 900°C in N_2. No significant differences are observed in the flake shape after thermal treatment.
